# Effect of humanine on the Notch signaling pathway in myocardial infarction

**DOI:** 10.55730/1300-0144.5734

**Published:** 2023-10-25

**Authors:** Elif ONAT, Ebru ÖNALAN, Berna ÖZDEM, Merve KAVAK BALGETİR, Tuncay KULOĞLU

**Affiliations:** 1 Department of Medical Pharmacology, Faculty of Medicine, Adıyaman University, Adıyaman, Turkiye; 2 Department of Medical Biology, Faculty of Medicine, Fırat University, Elazığ, Turkiye; 3 Department of Medical Biology and Genetics, Faculty of Medicine, İnönü University, Malatya, Turkiye; 4 In Vitro Fertilization Unit, Elazığ Fethi Sekin City Hospital, Elazığ, Turkiye; 5 Department of Histology and Embryology, Faculty of Medicine, Fırat University, Elazığ, Turkiye

**Keywords:** Myocardial infarction, humanin, Notch1, Hes1, Dll4

## Abstract

**Background/aim:**

By applying humanin (HN) before myocardial infarction (MI), its protection in myocardial injury and the possible roles of its cellular mechanism in the Notch pathway were investigated.

**Materials and methods:**

The study was carried out at Fırat University Experimental Research Center (12/24/2018–12/23/2019). Spraque-Dawley rats were divided into 10 groups: I (control) (n = 6), II (HN 6 h) (n = 6), III (HN 24 h) (n = 6), IV (HN day 7) (n = 6), V (MI 6 h) (n = 7), VI (MI 24 h) (n = 7), VII (MI day 7) (n = 7), VIII (MI+HN 6 h) (n = 7), IX (MI+HN 24 h) (n = 7), and X (MI+HN day 7) (n = 7). To create MI, 200 mg/kg of isoproterenol (ISO) was administered to the rats subcutaneously. Moreover, 252 μg/kg of HN was given intraperitoneally (ip) to the rats on its own and before MI. Molecular parameters Notch1, Notch2, Hes1, Hes2, Jagged1, Jagged2, DLL1, and DLL4 were examined using polymerase chain reaction in the heart tissue, Notch1, Hes1, and DLL4 were examined using western blot, while heart tissue was taken for histochemical examinations.

**Results:**

The mRNA expression levels of the Notch signaling members (Notch1, Notch2, Hes1, Hes2, Jagged1, Jagged2, DLL1, and DLL4) tended to decrease after MI. The Notch signaling members increased more significantly, especially toward day 7 after HN application before MI. In the western blot anylyses, the Notch1, Hes1, and DLL4 protein levels increased significantly toward day 7 in the groups given HN before MI. Moreover, the serum AST, LDH, CK-MB, and troponin I levels tended to decrease with the application of HN before MI and there was a significant decrease in edema, hemorrhage, and mononuclear cells in the heart tissue at 24 h post-MI and fibrosis on day 7 post-MI.

**Conclusion:**

HN administration before MI has a cardioprotective effect on rats via the Notch signaling pathway.

## 1. Introduction

Acute myocardial infarction (AMI) is still the primary reason for morbidity and mortality around the world. AMI occurs as a result of the obstruction of myocardial blood flow. It is triggered by coronary thrombosis and leads to myocardial cell death [1–3]. The pathogenesis of myocardial infarction (MI) includes the interaction of multiple mechanisms, such as cardiac overload, oxidative stress damage, impaired autophagy, and apoptosis, and these mechanisms cause heart damage [4,5]. Despite this information, the mechanisms related to heart damage are not fully understood. As a result of the administration of a cardioprotective pharmacological agent before ischemia, the heart can be protected against tissue, organ, or cell damage and severe ischemic attacks that may develop [6].

Isoproterenol (ISO) is a synthetic derivative of catecholamine employed to induce experimental MI in rats [7,8]. It is similar in structure to adrenaline. It affects β1 and β2 receptors and does not stimulate α receptors [9]. It has a vasoconstrictor effect at high doses and a vasodilator effect at low doses. The effects of ISO are due to its potent chronotropic and inotropic properties, increasing myocardial oxygen consumption, lowering diastolic arterial pressure and coronary perfusion pressure [10]. Using ISO in experimental MI induction for animals is a well-known model to investigate the effects of various compounds that have cardioprotective impacts.

It has been recently shown that a newly discovered peptide, humanin (HN), might have various useful effects, such as the reduction in cellular apoptosis, inflammation, and oxidative stress [11–13]. In physiological conditions, endogenous HN can be produced by various tissues, e.g., the brain, heart, skeletal muscle, and liver [14]. Endogenous HN levels are not sufficient to reduce myocardial damage if exposed to ischemia/reperfusion (I/R) injury [15]. However, the administration of a dose of 252 μg/kg of HN during ischemia may enhance the cardioprotective effects of endogenous HN in regard to myocardial I/R injury through increased HN levels in the affected myocardium [15]. Such evidence has shown that decreased endogenous HN levels may have important roles in the pathogenesis of cardiovascular diseases [16].

Many cellular processes are regulated by the Notch signaling pathway, including cell differentiation, proliferation, apoptosis, and regeneration. In mammals, 4 Notch receptors (Notch1–4) and 5 Notch ligands (Delta-like 1/3/4 and Jagged 1/2) have been described [17]. Notch receptor activation occurs by binding to Notch ligands. The target genes of Notch signaling, which include the basic helix-loop-helix transcription factors Hairy and enhancer of split 1 (Hes1) and Hairy-related transcription (HRT) factor family members exist in developing and adult hearts [17]. It is believed that Notch signaling has a key role in maintaining functional homeostasis in the adult heart, as demonstrated in studies attributed to cardiac patterns, such as MI, cardiac hypertrophy, and regurgitation [18].

It is known that various pharmacological agents have a protective effect due to their administration before/during ischemia or reperfusion. One of them, HN, is known to have a cardioprotective effect when applied before MI, but the effect mechanism of this cardioprotective effect on the Notch signaling pathway has not been fully understood. Therefore, this study aimed to investigate whether the Notch signaling pathway has a role in the cardioprotective effect of HN.

## 2. Materials and methods

### 2.1. Animals and experimental design

A total of 66 Spraque-Dawley rats (8–10 weeks old), weighing 200–210 g, supplied by Fırat University Experimental Research Center, were used in the study, in the same setting, and were provided standard water and food ad libitum. The study was carried out between 12/24/2018 and 12/23/2019. The rats were divided into 10 groups: group I (control) (n = 6), group II (HN 6 h) (n = 6), group III (HN 24 h) (n = 6), group IV (HN day 7) (n = 6), group V (MI 6 h) (n = 7), group VI (MI 24 h) (n = 7), group VII (MI day 7) (n = 7), group VIII (MI+HN 6 h) (n = 7), group IX (MI+HN 24 h) (n = 7), and group X (MI+HN day 7) (n = 7). While designating the groups, the study of Erten et al. [19] was taken as reference. The control group underwent no applications during the experiment, while 252 μg/kg of HN in saline was given to the rats in the HN-administered groups intraperitoneally (ip) [15]. HN (H6161, Sigma-Aldrich Corp., St. Louis, MO, USA) was administered to the rats in groups II, III, and IV. The rats in the other groups were administered 200 mg/kg of ISO (isoproterenol hydrochloride, I5627, Sigma-Aldrich Corp.) subcutaneously to induce MI [19]. HN was applied to the rats in groups VIII, IX, and X before ISO administration. The rats in groups II, V, and VIII at 6 h, in groups III, VI, and IX at 24 h, and in groups IV, VII, and X on day 7 were euthanized under anesthesia with ip ketamine (75 mg/kg) + xylazine (10 mg/kg). The thorax was opened and 5-cc intracardiac blood samples were drawn into biochemistry tubes. The anterior wall of the left ventricle of the heart was cut vertically into two halves. One half was fixed in 10% formaldehyde solution for histopathological examination, and the other half was stored at −80 °C for real-time polymerase chain reaction (RT-PCR) and western blot analyses.

### 2.2. Histochemical examination

Cardiac tissues of the groups were fixed in 10% formaldehyde for 24 h, irrigated for 24 h using tap water, underwent routine histological follow-up series, and embedded in paraffin blocks. Masson trichrome staining was applied on 5-μm sections of the paraffin blocks, which were examined with a Novel Optics N-800 M microscope (Nanjing Jiangnan Novel Optics Co., Ltd., Nanjing, Jiangsu, China) and photographed.

### 2.3. Polymerase Chain Reaction

For RNA isolation from the cardiac tissues, which were homogenized with a Bullet Blender Storm Pro homogenizer (Next Advance Inc., Troy, NY, USA), the TRIzol method was used [20]. The RNA samples were stored at −80 °C until use. RNA measurement (in μg/mL) was performed with an Oubit RNA Assay Kit and Oubit 2.0 Fluorometer (Invitrogen, Thermo Fisher Scientific Australia Pty Ltd., Scoresby, Australia). An RNA pool was prepared with the samples of the groups for each complementary DNA (cDNA) synthesis. For the cDNA synthesis, which was performed with a High-Capacity cDNA Reverse Transcription Kit (Applied Biosystems, Foster City, CA, USA) in a total volume of 20 μL, 2 μg of pooled RNA samples were used. The RT-PCR assays were made with rat-specific Notch1, Notch2, Hes1, Hes2, Jagged1, Jagged2, DLL1, and DLL4 (Cat No.: 190406; Sentegen Biotechnology, Ankara, Turkey), glyceraldehyde 3-phosphate dehydrogenase (GAPDH) Tag Man Assays (Applied Biosystems) and Tag Man Master (Applied Biosystems) mix in an ABI Prism 7500 Fast RT-PCR (Applied Biosystems) thermal cycler. The GAPDH gene was used as a control to determine the messenger RNA (mRNA) levels of the Notch1, Notch2, Hes1, Hes2, Jagged1, Jagged2, DLL1, and DLL4 genes. RT-PCR was applied in 3 replications and the differences in the gene expression were calculated using the 2-ΔΔCT method.

### 2.4. Western Blot

Protein extraction from the heart tissue samples was performed using lysis solution (4 μL of NAF to 1 mL of lysis solution and 4 μL of NA3VO4). A protein assay kit (DC Protein Assay Kit Cat. No.: 5000111; Bio-Rad, Hercules, CA, USA) was employed to determine the amount of total protein. Samples that contained 50 μg of total protein were transferred to polyvinylidene fluoride (PVDF) membranes by running them on 8% and 4%, and 12% and 6% sodium dodecyl sulfate-polyacrylamide gel (SDS-PAGE). For the gels, acrylamide (CAS 79-06-1; Acros Organics Bvba, Geel, Belgium), ammonium persulphate 98% (CAS 37727-54-0; Acros Organics Bvba), resolving buffer (Cat. No.: EC-893; National Diagnostics, Charlotte, NC, USA), sodium dodecyl sulfate (Cat. No.: BP166-500; Thermo Fisher Scientific, Inc., Waltham, MA, USA), stacking buffer (Cat. No.: EC-893; National Diagnostics), tris base (Cat. No.: 77-86-1; Sigma-Aldrich Chemical Co., St. Louis, MO, USA), tween 20 (Cat. No.: P1379; Sigma-Aldrich) were used. After blotting, the membranes were incubated overnight in Notch1, Hes1, and DLL4 polyclonal antibodies (respectively, 1:500, Cat. No.: BT-AP04681, BT-Lab, Shanghai Korain Biotech Co., Ltd., Shanghai, China; 1:200, Cat. No.: Fnab05799, fine test; 1:500, Cat. No.: Fnab02416, fine test; Cat. No.: BTAP03944, BT-Lab). The membranes, washed 3 times with tris-buffered saline/tween, were incubated in goat antirabbit secondary antibody (Cat. No.: AS0473, BT-Lab) for 1 h at room temperature. A solution of luminol and peroxide at a 1:1 ratio was used to view the bands. The UVP ChemiDoc-It2 imager (LabXMedia Group Inc, Midland, ON, Canada) was used for visualizing the blots and the densitometry measurements were made with ImageJ (US National Institutes of Health, Bethesda, MD, USA).

### 2.5. Serological analysis

Serum was obtained by centrifuging 5 cc of blood taken intracardially from each rat at 3000 rpm for 10 min. Serum samples for the biochemical analyses were stored at −80 °C until the day use. In order to confirm that MI developed in the rats, the aspartate aminotransferase (AST), creatine kinase-MB (CK-MB), lactate dehydrogenase (LDH), and troponin I levels, which are routinely measured parameters in MI, were examined.

Troponin I levels were measured using the immunoassay method on the ADVIA Centaur Analyzer (Siemens Healthcare Diagnostics Inc., Tarrytown, NY, USA). The CK-MB, LDH, and AST levels were measured using enzymatic methods on the ADVIA Chemistry Analyzer (Siemens Healthcare Diagnostics Inc.).

### 2.6. Statistical analysis

IBM SPSS Statistics for Windows 25.0 (IBM Corp., Armonk, NY, USA) was employed for the analyses. The data were given as the mean ± standard deviation (SD).

In terms of the normality analysis, the data exhibited a scattered pattern due to the fact that there were 3 samples in each group. The data were analyzed separately with the Kruskal–Wallis test, which is a nonparametric test for multiple group analysis, and one-way analysis of variance (ANOVA), which is a parametric test. As a result of these analyses, the same results were obtained in terms of significance. Due to the large number of groups and small number of samples, Bonferroni correction was the post hoc analysis method used, which can best distinguish the groups with significant differences. Bonferroni correction changes the probability (p) values to account for the increased probability of the type I error when testing with a large number of groups. The test allows for the comparison of several variables to prevent false data from appearing as statistically significant [21]. The results were evaluated with 95% confidence and 5% margin of error.

## 3. Results

### 3.1. Biochemical findings

As a result of HN administration, the serum AST, LDH and CK-MB levels showed a slight increase at 6 and 24 h post-MI compared to the control group but showed no change on day 7. The troponin I levels tended to be lower as a result of HN administration compared to the control (Table).

Serum AST and troponin I levels increased significantly at 6 and 24 h post-MI. As a result of MI, the LDH and CK-MB levels increased at all of the time intervals. The AST, LDH, CK-MB and troponin I levels were lower in the MI groups given HN, although it was not statistically significant (Table).

### 3.2. Histochemical findings (Masson trichrome staining)

**Figure 1:**
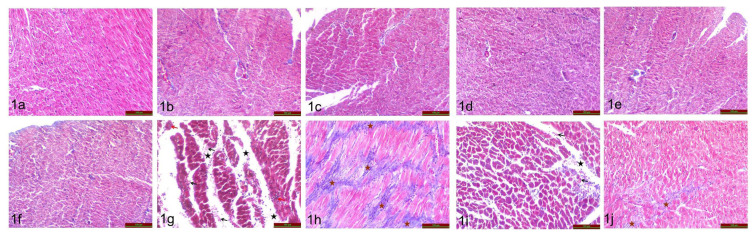
Histological findings of the cardiac tissues (Masson’s trichrome staining) for the whole duration [control (1a), HN 6 h (1b), HN 24 h (1c), HN day 7 (1d), MI 6 h (1e), MI 24 h (1g), MI day 7 (1h), MI+HN 6 h (1f), MI+HN 24 h (1i), and MI+HN day 7 (1j)] of observation.

As a result of the examination of the Masson trichrome-stained preparations of all of the groups under light microscopy, the control (Figure 1a), HN 6 h (Figure 1b), HN 24 h (Figure 1c), HN day 7 (Figure 1d), MI 6 h (Figure 1e), and MI+HN 6 h groups (Figure 1f) showed normal appearance.

Edema (black star), hemorrhage (black arrow), and mononuclear cell increase (red arrow) were observed at 24 h post-MI (Figure 1g) compared to control. On day 7 post-MI (Figure 1h) significant fibrosis (brown star) was observed.

Compared to the MI 24 h group, there was a significant reduction in edema (black star), hemorrhage (black arrow), and mononuclear cells (red arrow) in the MI+HN 24 h group (Figure 1i). Compared with the MI day 7 group, a significant reduction in fibrosis (brown star) was observed in the MI+HN day 7 group (Figure 1j).

### 3.3. RT-PCR findings

**Figure 2:**
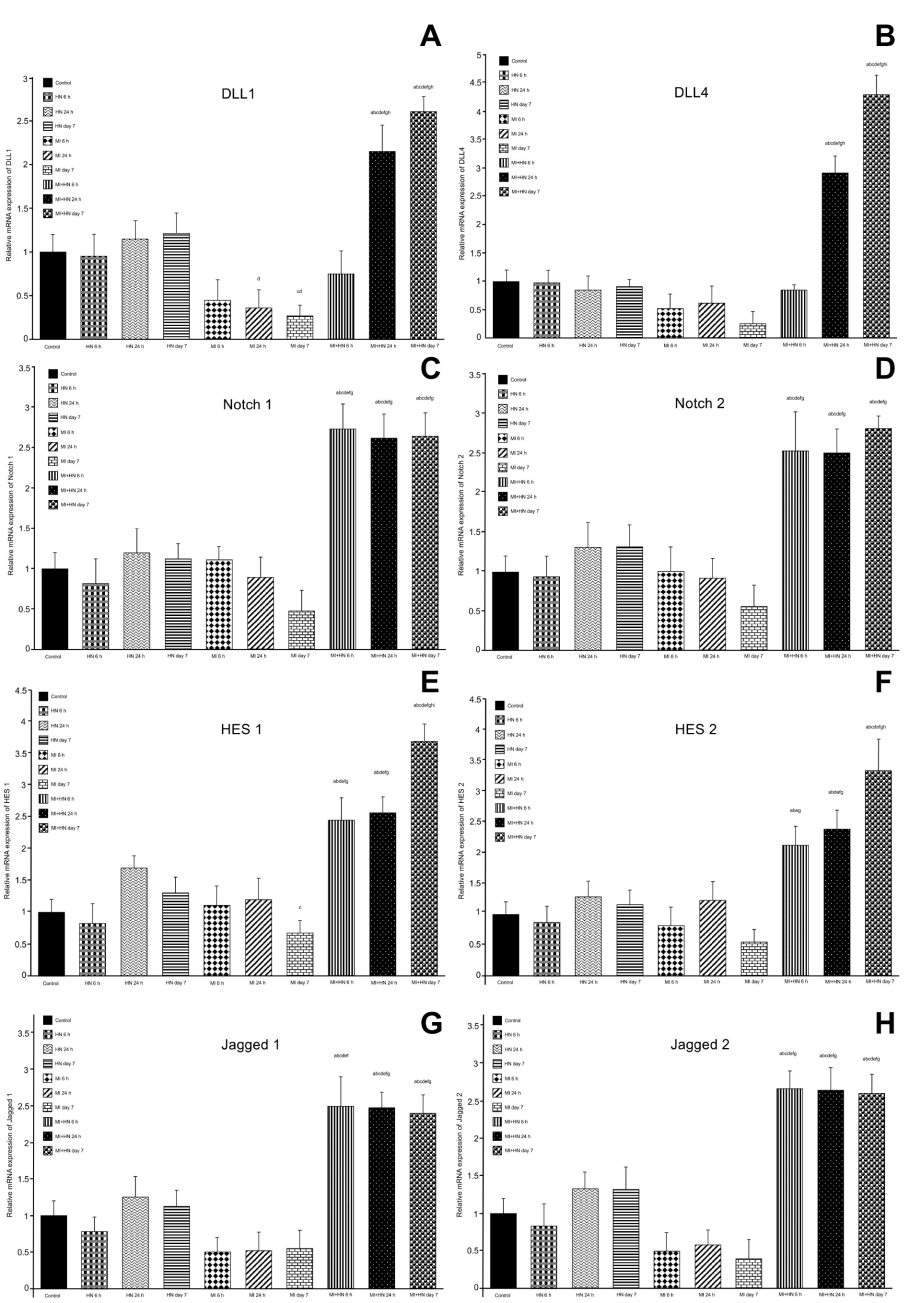
Levels of mRNA expression: a) p < 0.05 vs. the control. b) p < 0.05 vs. HN 6 h. c) p < 0.05 vs. HN 24 h. d) p < 0.05 vs. HN day 7. e) p < 0.05 vs. MI 6 h. f) p < 0.05 vs. MI 24 h. g) p < 0.05 vs. MI day 7. h) p < 0.05 vs. MI+HN 6 h (**A**). Levels of DLL4 mRNA expression: a) p < 0.05 vs. the control. b) p < 0.05 vs. HN 6 h. c) p < 0.05 vs. HN 24 h. d) p < 0.05 vs. HN day 7. e) p < 0.05 vs. MI 6 h. f) p < 0.05 vs. MI 24 h. g) p < 0.05 vs. MI day 7. h) p < 0.05 vs. MI+HN 6 h. i) p < 0.05 vs. MI+HN 24 h (**B**). Levels of Notch1 mRNA expression: a) p < 0.05 vs. the control. b) p < 0.05 vs. HN 6 h. c) p < 0.05 vs. HN 24 h. d) p < 0.05 vs. HN day 7. e) p < 0.05 vs. MI 6 h. f) p < 0.05 vs. MI 24 h. g) p < 0.05 vs. MI day 7 (**C**). Levels of Notch2 mRNA expression: a) p < 0.05 vs. the control. b) p < 0.05 vs. HN 6 h. c) p < 0.05 vs. HN 24 h. d) p < 0.05 vs. HN day 7. e) p < 0.05 vs. MI 6 h. f) p < 0.05 vs. MI 24 h. g) p < 0.05 vs. MI day 7 (**D**). Levels of Hes1 mRNA expression: a) p < 0.05 vs. the control. b) p < 0.05 vs. HN 6 h. c) p < 0.05 vs. HN 24 h. d) p < 0.05 vs. HN day 7. e) p < 0.05 vs. MI 6 h. f) p < 0.05 vs. MI 24 h. g) p < 0.05 vs. MI day 7. h) p < 0.05 vs. MI+HN 6 h. i) p < 0.05 vs. MI+HN 24 h (**E**). Levels of Hes2 mRNA expression: a) p < 0.05 vs. the control. b) p < 0.05 vs. HN 6 h. c) p < 0.05 vs. HN 24 h. d) p < 0.05 vs. HN day 7. e) p < 0.05 vs. MI 6 h. f) p < 0.05 vs. MI 24 h. g) p < 0.05 vs. MI day 7. h) p < 0.05 vs. MI+HN 6 h (**F**). Levels of Jagged1 mRNA expression: a) p < 0.05 vs. the control. b) p < 0.05 vs. HN 6 h. c) p < 0.05 vs. HN 24 h. d) p < 0.05 vs. HN day 7. e) p < 0.05 vs. MI 6 h. f) p < 0.05 vs. MI 24 h. g) p < 0.05 vs. MI day 7 (**G**). Levels of Jagged2 mRNA expression: a) p < 0.05 vs. the control. b) p < 0.05 vs. HN 6 h. c) p < 0.05 vs. HN 24 h. d) p < 0.05 vs. HN day 7. e) p < 0.05 vs. MI 6 h. f) p < 0.05 vs. MI 24 h. g) p < 0.05 vs. MI day 7 (**H**).

Notch signaling activation was measured using RT-PCR. There was an overall trend of increased levels of Notch signaling members, except for DLL4, at 24 h and 7 days post-MI in the groups given only HN. There was a general decreasing trend in the levels of Notch signaling members at all of the time intervals in the MI groups (Figures 2A–2H). The DLL1 and DLL4 mRNA expression levels were significantly increased at 24 h and 7 days post-MI in the MI+HN groups (Figures 2A and 2B). The levels of Notch1, Notch2, Hes1, Hes2, Jagged1, and Jagged2 were increased at significant levels in all h of the MI groups given HN (Figures 2C–2H).

### 3.4. Western blot findings

**Figure 3:**
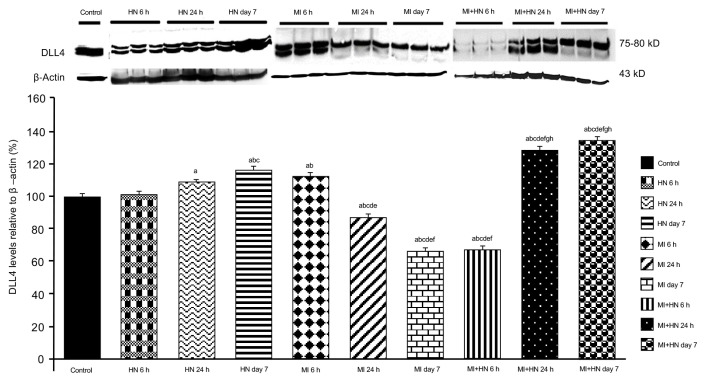
Relative DLL 4 protein expression (%) between the groups. β-Actin was used as the reference protein. a) p < 0.05 vs. the control. b) p < 0.05 vs. HN 6 h. c) p < 0.05 vs. HN 24 h. d) p < 0.05 vs. HN day 7. e) p < 0.05 vs. MI 6 h. f) p < 0.05 vs. MI 24 h. g) p < 0.05 vs. MI day 7. h) p < 0.05 vs. MI+HN 6 h.

**Figure 4:**
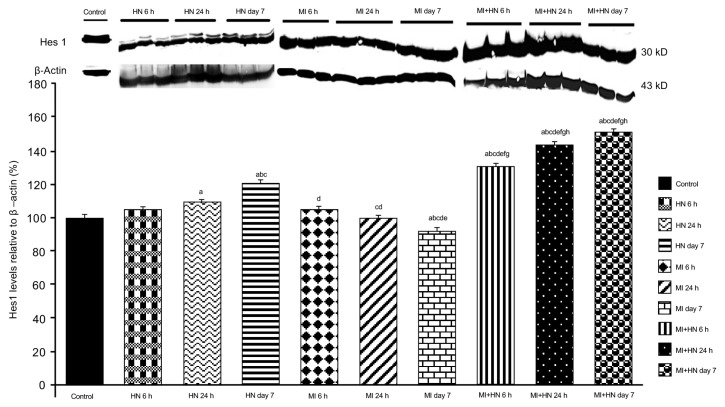
Relative Hes1 protein expression (%) between the groups. β-Actin was used as the reference protein. a) p < 0.05 vs. the control. b) p < 0.05 vs. HN 6 h. c) p < 0.05 vs. HN 24 h. d) p < 0.05 vs. HN day 7. e) p < 0.05 vs. MI 6 h. f) p < 0.05 vs. MI 24 h. g) p < 0.05 vs. MI day 7. h) p < 0.05 vs. MI+HN 6 h.

**Figure 5:**
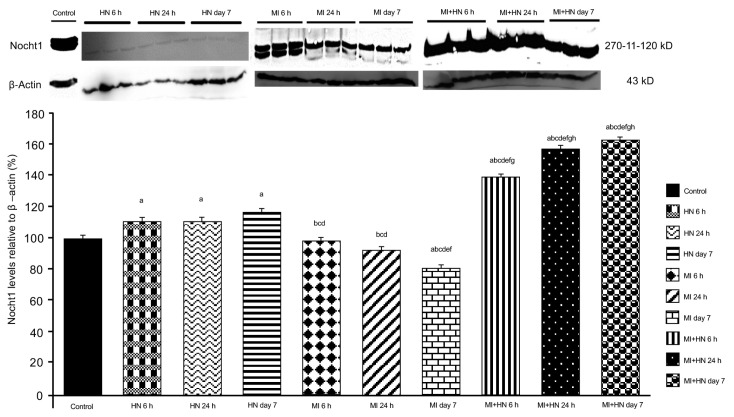
Relative Notch1 protein expression (%) between the groups. β-Actin was used as the reference protein. a) p < 0.05 vs. the control. b) p < 0.05 vs. HN 6 h. c) p < 0.05 vs. HN 24 h. d) p < 0.05 vs. HN day 7. e) p < 0.05 vs. MI 6 h. f) p < 0.05 vs. MI 24 h. g) p < 0.05 vs. MI day 7. h) p < 0.05 vs. MI+HN 6 h.

When compared to the control, an increase was detected in the levels of Notch signaling members, expecially in the HN 24 h and HN day 7 groups (Figures 3–5). A decrease was observed in the levels of DLL4 and Hes1 at 24 h and day 7 post-MI (Figures 3 and 4). Notch1 levels were decreased at all of the time intervals in the MI groups (Figure 5). The DLL4 level was lower in the MI+HN 6 h group when compared to the MI groups and it increased significantly in the MI+HN 24 h and MI+HN day 7 groups when compared to the MI groups. The Hes1 and Notch1 levels were significantly increased at all of the time intervals in the MI+HN groups compared to the MI groups.

## 4. Discussion

Although the protective effect of HN on myocardial damage has been demonstrated by in vivo and in vitro studies, the mechanisms by which HN exerts this protective effect are not fully known. As a result of this study, it has been shown for the first time that the Notch signaling pathway may contribute to this protective effect, as demonstrated by the biochemical and histological findings that HN has a cardioprotective effect when administered prior to MI.

Previous studies have shown that the addition of HN (84 μg/kg) before ischemia can protect against myocardial I/R injury [16]. A study conducted by Muzumdar [12] showed that administration of high-dose HN (2 mg/kg) by intracardiac injection in reperfusion may provide cardioprotection against I/R injury, indicating that high-dose HN may be beneficial. There was a 37% reduction in the MI area in the cardiac tissue of I/R rats treated with 252 μg/kg HN administration during ischemia, compared to vehicle-treated I/R rats [15]. These findings showed that HN can reduce the infarct area in mice with I/R, which is consistent with a previous study, although the routes of drug administration were different (intracardiac, intravenous) [12]. In physiological conditions, endogenous HN levels are not sufficient to reduce myocardial damage when exposed to I/R injury. However, the administration of a dose of 252 μg/kg HN in during ischemia may enhance the cardioprotective effects of endogenous HN against myocardial I/R injury through increased levels of HN in the affected myocardium [15]. The fact that the effect of HN against I/R damage is greater if given during ischemia than at the beginning of reperfusion at the same dose reveals the importance of HN administration at the onset of myocardial ischemia [15]. In the current study, reduced pathological changes such as edema, hemorrhage, mononuclear cells, and fibrosis observed in the histological examination of the heart tissue of rats in which MI was induced by applying ISO once again showed the cardioprotective effect of HN. Consistent with these findings, the increase in serum AST, LDH, CK-MB, and troponin I levels, which occur with MI, tended to decrease after HN administration, which supported the results herein.

The myocardial I/R injury process is thought to involve several cellular signaling pathways [22,23]. Some studies have reported that the Notch signaling pathway has a role in the regulation of cardiomyocyte proliferation, myocardial trabeculation, valve formation, and maintenance of adult heart tissue integrity. Especially Notch1 is an important adaptive signaling pathway member in I/R damage to various organs (e.g., the heart) [17]. Activation of the Notch1 gene in H9c2 cells was shown to support cell proliferation, inhibiting apoptosis in ischemic postconditioning [14]. In a consistent manner, Notch1 can protect cardiomyocytes against hypoxia or I/R injury [17]. In myocardial I/R injury, these findings support the idea that Notch activation is a favorable signal. Notch1 is an important receptor of the Notch signaling pathway, which is involved in various hypoxic states such as myocardial I/R injury [23,24,26]. Activation through ligands is usually detected on the surface of neighboring cells. The Notch receptor undergoes intramembrane proteolysis, and an intracellular area is exposed in the nucleus, resulting in signal transduction [24,25]. These then form a transcriptional activator complex initiating transcription of target genes in Notch efflux (e.g., Hes1) [22]. However, growing evidence shows that the reactivation of Notch signaling can induce an adaptive response for tissue regeneration or increased survival after pathological stress [17]. For example, endogenous Notch activation following MI in adults may support antiapoptosis, increase survival rates, and improve cardiac performance [27]. A previous study reported that Notch1 signaling activation in H9c2 cardiomyoblasts confers cardioprotection through ischemic preconditioning and postconditioning [28]. Pei et al. [29] showed that Notch1 signaling protects against reperfusion injury in ischemic mouse myocardium. In the current study, the level of Notch signaling members tended to decrease after MI, increase in the HN groups, and increase significantly in the MI+HN groups, demonstrating the key role of Notch signaling in the heart to maintain functional homeostasis. This was also confirmed by the observation of an increase in Notch1, Hes1, and DLL4 protein levels toward day 7 in the groups given HN. This indicated that HN may have a protective effect in MI, particularly on fibrosis and the Notch signaling pathway may mediate this effect. At the same time, this result proved that the protective effect of HN is more pronounced in the presence of myocardial damage.

The most important limitations of this study were that the findings were not supported by imaging methods such as electrocardiogram/echocardiography. Another important limitation was that a limited number of rats were used. It is recommended that future research studies with a larger number of rats should be conducted to test the reliability of the findings.

In conclusion, the cardioprotective effect of HN was demonstrated by biochemical and histological findings and it was seen that the Notch signaling pathway may contribute to this protective effect.

## Figures and Tables

**Table:** ELISA findings.

Groups	AST (U/L)	LDH (U/L)	CK-MB (U/L)	Troponin I (μg/L)
Control	127 ± 4.70	325.25 ± 18.55	625.42 ± 28.23	0.27 ± 0.49
HN 6 h	140	385	700	0.03
HN 24 h	131 ± 1.41	354.5 ± 6.36	647.5 ± 3.53	0.003 ± 0.004
HN day 7	126.5 ± 2.12	322 ± 7.07	627.5 ± 3.53	0
MI 6 h	451.37 ± 159.34abcd	1263.12 ± 319.23acd	1112.7 ± 117.74abcd	10.26 ± 2.81acd
MI 24 h	419.71 ± 301.36acd	1183.57 ± 363.7acd	967.5 ± 120.17acd	8.84 ± 5.37acd
MI day 7	214.12 ± 35.71e	1041.62 ± 122.48acd	865.64 ± 284.82ade	0.009ef
MI+HN 6 h	367.83 ± 109.30acdg	1186.67 ± 430.37acd	817.78 ± 125.87ade	7.52 ± 1.95acdg
MI+HN 24 h	362.12 ± 93.54acdg	888.5 ± 191.8ae	796 ± 151.54ae	4.57 ± 3.37acd
MI+HN day 7	192.8 ± 54.98efhi	810.7 ± 193.34de	738.2 ± 65.45e	0.001 ± 0.002efhi

Error bars indicate the SD. a) p < 0.05 vs. the control. b) p < 0.05 vs. HN 6 h. c) p < 0.05 vs. HN 24 h. d) p < 0.05 vs. HN day 7. e) p < 0.05 vs. MI 6 h. f) p < 0.05 vs. MI 24 h. g) p < 0.05 vs. MI day 7. h) p < 0.05 vs. MI+HN 6 h. i) p < 0.05 vs. MI+HN 24 h.
